# The *Methylococcus capsulatus* (Bath) Secreted Protein, MopE*, Binds Both Reduced and Oxidized Copper

**DOI:** 10.1371/journal.pone.0043146

**Published:** 2012-08-20

**Authors:** Thomas Ve, Karina Mathisen, Ronny Helland, Odd A. Karlsen, Anne Fjellbirkeland, Åsmund K. Røhr, K. Kristoffer Andersson, Rolf-Birger Pedersen, Johan R. Lillehaug, Harald B. Jensen

**Affiliations:** 1 Department of Molecular Biology, University of Bergen, Bergen, Norway; 2 Department of Chemistry, Norwegian University of Science and Technology, Trondheim, Norway; 3 Norwegian Structural Biology Centre, Faculty of Science, University of Tromso, Tromso, Norway; 4 Department of Molecular Biosciences, University of Oslo, Oslo, Norway; 5 Department of Earth Science–Centre for Geobiology, University of Bergen, Bergen, Norway; 6 School of Chemistry and Molecular Biosciences, The University of Queensland, Brisbane, Australia; Griffith University, Australia

## Abstract

Under copper limiting growth conditions the methanotrophic bacterium *Methylococcus capsulatus* (Bath) secrets essentially only one protein, MopE*, to the medium. MopE* is a copper-binding protein whose structure has been determined by X-ray crystallography. The structure of MopE* revealed a unique high affinity copper binding site consisting of two histidine imidazoles and one kynurenine, the latter an oxidation product of Trp130. In this study, we demonstrate that the copper ion coordinated by this strong binding site is in the Cu(I) state when MopE* is isolated from the growth medium of *M. capsulatus*. The conclusion is based on X-ray Near Edge Absorption spectroscopy (XANES), and Electron Paramagnetic Resonance (EPR) studies. EPR analyses demonstrated that MopE*, in addition to the strong copper-binding site, also binds Cu(II) at two weaker binding sites. Both Cu(II) binding sites have properties typical of non-blue type II Cu (II) centres, and the strongest of the two Cu(II) sites is characterised by a relative high hyperfine coupling of copper (A_||_ = 20 mT). Immobilized metal affinity chromatography binding studies suggests that residues in the N-terminal part of MopE* are involved in forming binding site(s) for Cu(II) ions. Our results support the hypothesis that MopE plays an important role in copper uptake, possibly making use of both its high (Cu(I) and low Cu(II) affinity properties.

## Introduction

Copper is essential for most living organisms, and tight homeostatic regulation of copper acquisition, distribution, and use is generally required. Copper appears to play a central role in the physiology of methanotrophs by controlling the ability of these cells to utilize methane as their carbon and energy source (for recent reviews see [1] and [2]). *Methylococcus capsulatus* (Bath) belongs to a small subset of methanotrophs that can produce a soluble methane monooxygenase (sMMO) in addition to the membrane-bound copper enzyme particulate methane monooxygenase (pMMO) [3], [4]. These enzymes catalyze the oxidation of methane to methanol, the initial and obligate step for carbon fixation and energy production. One of the most important factors controlling methanotrophic activity is the Cu-to-biomass ratio. pMMO is dependent on copper availability of both Cu(I) and Cu(II) for expression and catalytic activity [5]. sMMO, containing a diiron active site, does not require copper for catalytic activity, and is only produced when the copper level in the growth environment is low.

Evidence suggests that methanotrophs have an active copper uptake system [1], [3], [6], and thus their copper-homeostatic activity differs from that of other prokaryotes in which systems handling extra-cellular copper is mainly focused on detoxification and elimination [7]. Methanobactin has been implicated in copper sensing and uptake in several methanotrophs [8], [9], [10], [11], [12], [13], (reviewed in [1], [14]). In the case of *Methylosinus trichosporium* OB3b, there is ample evidence that this copper-binding siderophore-like molecule is the (extracellular) component of a Cu uptake system (reviewed in [1]). In *M. capsulatus*, both methanobactin and MopE have been proposed to be involved in copper uptake [1], [14], [15], [16], [17].

**Figure 1:**
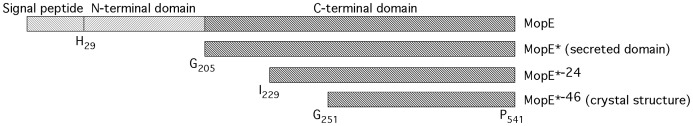
Structural organization of the MopE protein.

MopE consisting of 541 amino acids was first identified as one of five outer membrane-associated proteins designated MopA-E [18]. Later it was found that MopE is expressed under copper limiting conditions, and is both located on the cell surface (MopE^C^) and secreted into the growth medium (MopE*). MopE^C^ corresponds to full-length MopE, while the secreted MopE* is truncated at the N-terminus and contains only the last 336 amino acids of MopE^C^ (Fig. 1) [19]. The properties of MopE indicate that it may play a role in copper homeostatic activities: It is cell surface located, secreted to the medium, is down-regulated by copper and has a high affinity for copper. In particular, quantitative analysis by inductively coupled plasma mass spectrometry (ICP-MS) [20] showed that MopE* purified from *M. capsulatus* (Bath) cells grown in NMS medium without added copper ions (copper depleted medium) was found to contain about 0.6 copper ions per protein molecule. The same ratio was estimated from the electron density maps of the crystal structure of MopE* [20]. Competition experiments using Bathocuproine, a copper chelator, indicated that this copper ion was bound strongly to the protein (*K*_d_<10^−20^M) [20]. The X-ray structures of wild type MopE* [20] revealed that the wild type MopE* contained a partially buried copper ion in a distorted tetrahedral site consisting of an oxygen ligand from a water molecule, two histidine imidazoles (His 132 and His203), and at the fourth position, the N1 atom of kynurenine, an oxidation product of Trp130 [20]. These data revealed for the first time the involvement of the tryptophan metabolite kynurenine in a protein metal-binding site.

This study presents further evidence about the importance of MopE* in copper uptake and handling in *M. capsulatus* (Bath). We show by Electron Paramagnetic Resonance (EPR) and X-ray Near Edge Absorption Spectroscopy (XANES) that MopE* binds both reduced and oxidized copper ions. The strong copper binding site, identified by crystal structure studies [20], binds copper in the reduced (Cu(I)) state (Fig. S1), whereas two Cu(II) binding sites have significantly lower affinities., i.e. in the µmolar range. Immobilized metal ion affinity chromatography (IMAC) experiments indicated that amino acid residues within the 24 first N-terminal residues of MopE* are involved in defining these Cu(II) binding site. Our results support the hypothesis that MopE plays an important role in copper uptake, presumably utilizing both its high Cu(I) and low Cu(II) affinity properties.

## Experimental

### Growth Conditions and Purification of MopE* from Spent Medium

MopE* was purified using copper-free buffer consisting of 20 mM Tris pH 7,5 80 mM NaCl and 1 mM CaCl_2_ (20) as described [20] from spent medium of *M. capsulatus* (Bath) strain NCIMB 11132 grown in continuous cultures in nitrate mineral salt (NMS) medium [21] without added copper [16]. The purity and stability of purified MopE* was assessed by SDS/PAGE analysis [22] using 10% (w / v) running gels and 3% (w / v) stacking gels.

### Determination of Protein Concentration

The concentration of MopE* was determined by UV/VIS spectroscopy using the absorbance at 280 nm. A UNICAM UV/ VIS UV2 spectrometer supplied with a 1 cm path length quartz cuvette was used for the measurements. The molar extinction coefficient for MopE* was estimated from its amino acid composition to be 77475 cm^−1^ by the ExPASy Protparam tool (http://us.expasy.org/tools/protparam.html). Alternatively, the concentration of MopE* was determined by ICP-MS, based on the sulphur signal. The protein concentration of MopE* determined by either ICP-MS or the absorbance at 280 nm differed by less then 10%. Furthermore, analysis using solutions of BSA (Sigma) and Beta lactoglobulin (Sigma) gave protein concentrations based on ICP-MS data with an accuracy better than 90% compared to the theoretical calculated concentrations.

### Inductively Coupled Plasma Mass Spectrometry (ICP-MS)

The copper content of MopE* was determined by Inductively Coupled Plasma Mass Spectrometry (ICP-MS) at the Center for Element and Isotope Analyses (CEIA), University of Bergen, Norway. Prior to analysis, the samples were hydrolysed with nitric acid (6% v/v) overnight on a hotplate (110°C). A single collector double focusing magnetic sector field ICP-MS spectrometer (Finnigan Element 2) was used for the copper analyses. The samples were diluted in 2% HNO_3_ and analysed by the standard addition method using an ICP multi-element standard (Merck # 1.0580.0100) for calibration. Oyster Tissue Standard (NIST 1566a) was used as an external reference standard.

### Electron Paramagnetic Resonance (EPR)

X-band EPR analyses of MopE* were performed with a Bruker Elexsys 500 EPR spectrometer fitted with an Oxford ESR 900 helium flow cryostat, a Bruker ER4116DM dual mode cavity, or a Super X kv319 cavity. The temperature was set to 33 K for the Cu(II) titration, but some samples were examined between 4–77 K using microwave powers from 1 microW up to 100 milliW. No difference in microwave power saturation behaviour of the Cu(II) EPR signals were detected at temperatures 33–77 K in presence of different amounts added Cu(II) to MopE*. At ∼77 K a cold finger devise was used with an EPR tube immersed in liquid nitrogen. Each experiment consisted of MopE* at a concentration of 360 µM in a buffer consisting of 20 mM MOPS pH 7.5, 80 mM NaCl, 1 mM CaCl_2_, and the desired concentration of CuCl_2_, and samples were incubated at 22°C for 10 min prior to freezing in liquid nitrogen The operating parameters are given in the figure legends. The concentration of EPR active copper in each experiment was determined by double integration using the software WinEPR from Bruker and comparison to either a 0.2 or 1 mM Cu(II) in 1 M HClO_4_ [23] or 0.2 or 1 mM Cu(II)EDTA complex as standards under non microwave power saturating conditions.

### X-Ray Absorption Spectroscopy (XAS) Data Collection

X-ray absorption data were collected in the fluorescence mode at the copper K-edge at the Swiss Norwegian Beamlines (SNBL, BM01b) using a 13 element Ge multi–channel detector. The MopE* (0.7 mM) in buffer solution consisting of 20 mM MOPS pH 7.5, 80 mM NaCl, 1 mM CaCl_2_ was filled in a Perspex sample holder with kapton windows yielding a sample thickness of 2.5 mm. Spectra were measured with 5 eV steps below the edge, 0.2 eV steps in the edge region, and steps equivalent to 0.04 Å^−1^ increments above the edge (region borders were 8960, 9030, and 9060 eV). Several XAS scans were collected and summed. All XANES spectra were energy corrected against a copper-foil calibration (8979 eV).

### XAS Analysis

The XAS data were summed and background subtracted using the Athena program [24]. The edge energy, E_o_, was determined at the first inflection point after the pre-edge using the derivative spectra. The peak fitting procedures contained in the Athena program were used to determine the pre-edge positions. For the pre-edge peak fitting an arctangent function was used to model the step portion of the data with the centroid value set to the Eo value before refinements. The Eo value was determined as the first inflection point after the pre-edge in all cases. The fitting range was manually chosen, and then varied to give the optimal fit. The pre-edge peak centroid was determined manually and then refined. Both the Gaussian and Lorenztian functions were used, but only the former gave conclusive fits.

XANES data were collected of Cu(I) oxide [25], Cu(I) diamine ([Cu(NH_3_)_2_]^+^) and Cu(II) tutton salt (Cu(NH_4_)_2_(SO_4_)_2_⋅6H_2_O) [26] to use as references. The Cu(I) diamine solution was prepared essentially as described in [27].

### Copper Binding Experiments

#### Equilibrium dialysis

Dialysis of MopE* against various concentrations of CuCl_2_ was conducted to investigate the binding of Cu(II) under equilibrium conditions. Dialysis cassettes (molecular-mass cut-off of 10 kDa), filled with 500 µL protein suspension (10 µM), were placed into 100 mL of 20 mM Tris-HCl pH 7.5, 80 mM NaCl, 1 mM CaCl_2_ containing CuCl_2_ at concentrations ranging from 0 to 100 µM. Dialysis was carried out over night at 4°C under constant stirring. The Cu concentrations inside and outside the dialysis cassettes were determined by ICP-MS as described above. The data were adjusted for copper bound to MopE* in copper free buffer.

#### Immobilized metal ion affinity chromatography (IMAC)

A Chelating SepharoseTM Fast Flow kit (Amersham Biosciences) was used according to the manufacturer’s description. Cu(II) ions were immobilized on the Sepharose via iminodiacetic acid (IDA), and a 0.5 ml column was charged with 0.2 M CuCl_2_ and equilibrated using 20 mM sodium phosphate buffer pH 6.8, containing 0.5 M NaCl. Following the same procedure, columns were also prepared using other metal ions, in particular Fe(II), Fe(III), Ni(II), Zn(II), and Co(II). 1.5 ml of concentrated spent medium from *M. capsulatus* was applied to each column; the flow through collected, and the column washed with three column volumes with 20 mM sodium phosphate buffer pH 6.8, containing 0.5 M NaCl and 1 mM CaCl_2_. Elution was performed using three column volumes 20 mM sodium phosphate buffer pH 6.0, containing 0.5 M NaCl and 1 mM CaCl_2_, followed by four column volumes of the same buffer adjusted to pH 4.0. The column retained its blue colour, indicating that the iminodiacetate-bound copper has reasonable stability at pH 4.0 [28]. Alternatively, elution could be performed using 100 mM imidazole. Both the wash and elution fractions were analyzed by SDS-PAGE. A degradation product of MopE* identified in the wash fractions was characterized using N-terminal sequencing at the protein sequencing facility at the University of Oslo.

## Results

### Cu(I) is Bound in a High Affinity Site of MopE

MopE was purified from spent media as described previously (20). CaCl_2_ was included in the buffer, because during optimization of the purification conditions we discovered that the presence of calcium ions increased the stability of the protein by preventing proteolytic degradation. The copper content of purified MopE* was determined by ICP-MS to be 0.6 copper ions per protein molecule. Prior to the EPR analysis MopE* was dialysed against a MOPS buffer (20 mM MOPS pH 7.5, 80 mM NaCl and 1 mM CaCl_2_) since the pKa of Tris show a strong temperature dependence [29] inducing freezing artefacts, and the EPR studies were performed at low temperatures (4–77 K).

**Figure 2:**
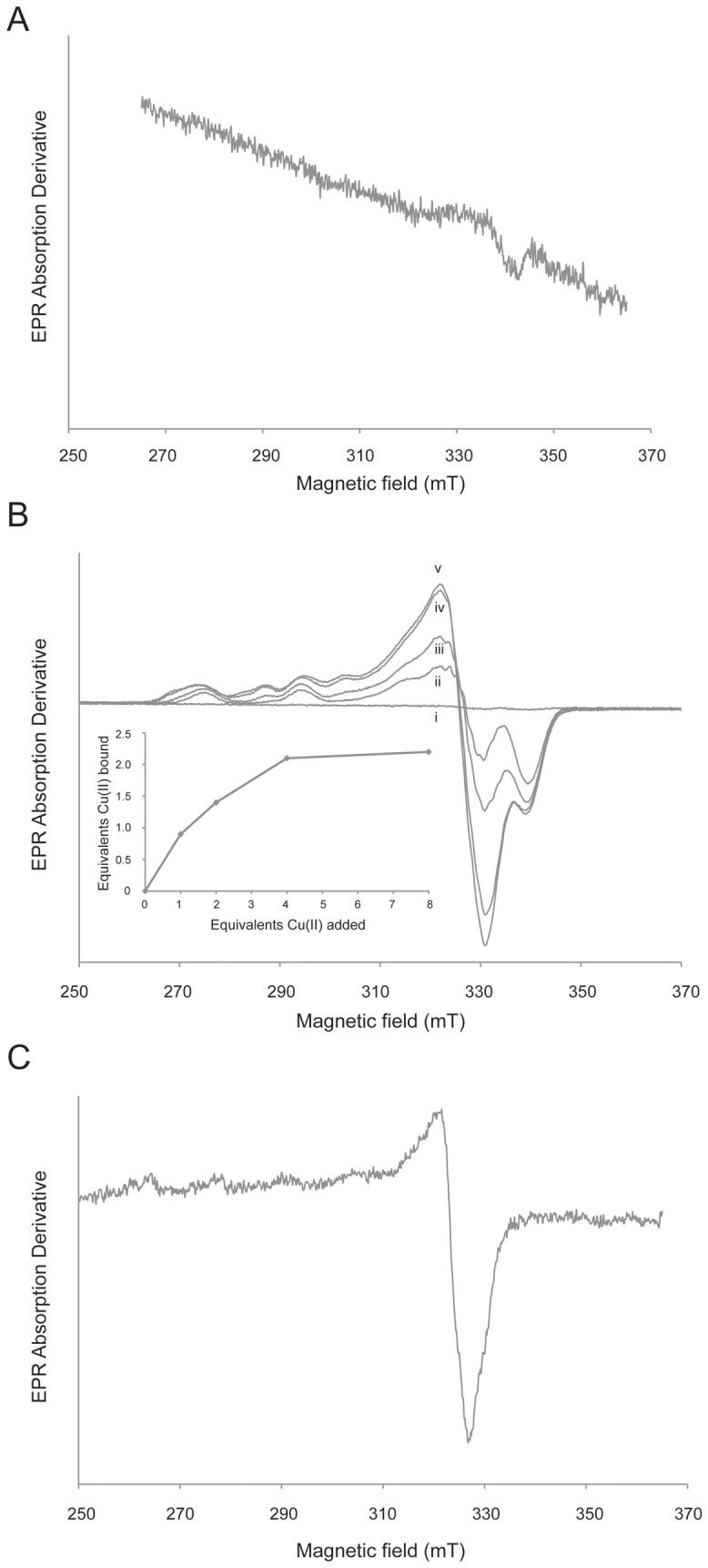
EPR analysis of MopE*. A) EPR spectrum of Mops buffer with 1 mM CuCl_2_. The spectrum was recorded at a temperature of 77 K with a modulation frequency of 100 kHz, a modulation amplitude of 1.0 mT; and a time constant of 164 ms. The microwave frequency was 9.57 GHz, and the microwave power was 1 mW. B) EPR spectra of MopE* (360 uM) as purified (i), and with 1, 2, 4, and 8 (ii–v) molar equivalents of Cu(II) respectively. Copper was added as CuCl_2_ from a freshly prepared solution in water. The spectra were recorded at 33 K, a modulation frequency of 100 kHz, a modulation amplitude of 0.6 mT, a time constant of 41 ms, a microwave frequency of 9.37 GHz, and a microwave power of 0.1 mW. The inset shows EPR-detected Cu(II) as a function of added Cu(II), demonstrating near saturation after addition of 4 molar equivalents of Cu(II). C) EPR spectrum of MopE* after treatment with 2.5% nitric acid. The spectrum was recorded at a temperature of 27 K, a modulation amplitude of 0.6 mT, a time constant of 40,960 ms, a microwave frequency of 9.39 GHz, and a microwave power of 0.05 mW.

The EPR spectrum of purified MopE* (360 uM) in MOPS buffer did not present any characteristic signal of Cu(II) without added CuCl_2_, indicating that the copper bound to the purified protein is in the Cu(I) state (Fig. 2B, curve i). We examined the protein between 4K and 77K without detecting any EPR active signal. The EPR spectrum of MopE* crystals (about 100–200 crystals in 200 µl 45% ammonium sulphate and 0.1 M Hepes pH 7.5) was also recorded, and this showed no signal characteristic for Cu(II). Oxidation attempts with 2 mM hydrogen peroxide did not increase EPR active Cu(II) in MopE* in solution or crystals, while 2.5% of nitric acid treatment of MopE* at room temperature prior to EPR analysis generated an EPR active Cu(II) signal. A maximum of 0.4±0.15 copper ions was recovered per protein molecule in solution (Fig. 2C), supporting the presence of copper as Cu(I) in the purified MopE*. It is possible that not all of the Cu ions have been released from the protein or oxidized during nitric acid treatment. This may explain the observed discrepancy in the copper to protein ratio of 0.6 and 0.4 calculated from the ICP-MS data and the EPR based quantification, respectively.

**Figure 3:**
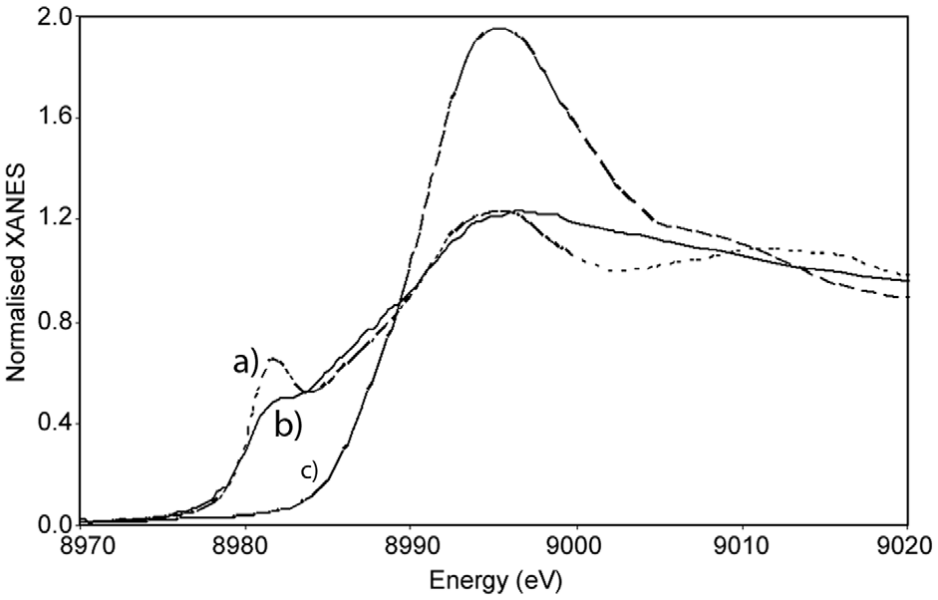
XAS analysis of MopE*. Normalised XANES of a) Cu(I) oxide (–), b) MopE* protein (–), and c) Cu(NH_4_)_2_(SO_4_)_2_⋅6H_2_O (Cu(II) tutton salt) (–).

The XANES analysis of MopE* was also in line with copper being monovalent in the protein (Fig. 3). The position of the edge energy gives information regarding the oxidation state [30]. MopE* exhibited a pre-edge feature at 8982.1 eV attributed to the 1s4p transition. The position of the feature is shifted to higher energies compared to that seen for Cu(I) oxide, 8981.3 eV (Fig. 3), and lower energies compared to that reported previously for Cu(I) centres in methane monooxygenase (8983–84 eV) [5] and Cu+-ATPases (8984 eV) [31]. However, the position of the pre-edge feature is comparable to that of the Cu(I) model compound, Cu(I) diamine in solution (8982.8 eV) (not shown). In addition (Fig. 3), the edge position (8985 eV) is substantially lower than that observed for the Cu(II) model compound Cu-tutton (8990–91 eV) clearly supporting the presence of monovalent copper in the MopE* protein. The intensity of the pre-edge feature at 8982 eV is relatively low in MopE*, precluding a linear and two-coordinate Cu(I) structure in the protein, thus suggesting either three or four coordinate Cu(I) environments in the protein [30], in line with the crystal structure of the protein [20].

### MopE* has Two Cu(II) Binding Sites

The EPR spectra recorded of MopE* (with Cu(I) bound) when incubated for 10 min at 22°C in MOPS buffer containing 1, 2, 4, or 8 mol equivalents of CuCl_2_ are presented in Fig. 2B, lines ii–v. For each titration point the total amount of EPR active Cu(II) was calculated by comparing the double integral of the first-derivative EPR signal to a standard consisting of 0.2 mM Cu(II) in 1 M HClO_4_. The total amount of EPR active Cu(II) was then plotted as a function of the total amount of Cu(II) added to the sample (inset Fig. 2B). Figure 2A shows the control spectrum of the MOPS buffer with 1 mM CuCl_2_ at 77 K, showing no EPR signal. Spectra of this sample were also recorded at 10 and 33 K and no EPR signal could be observed (results not shown). This is consistent with a report by the Van Doorslaer group, which showed that EPR signals of 0.3 and 2 mM Cu(II) in MOPS buffer could not be observed [32]. In water, dipolar broadening of Cu(II) EPR spectra have been observed [33], [34] and at a pH >7, EPR silent aquo copper are readily formed [32], [35]. Since MOPS possesses no, or a very low, affinity for copper [36], these observations may explain the lack of a Cu(II) EPR signal in the control.

**Figure 4:**
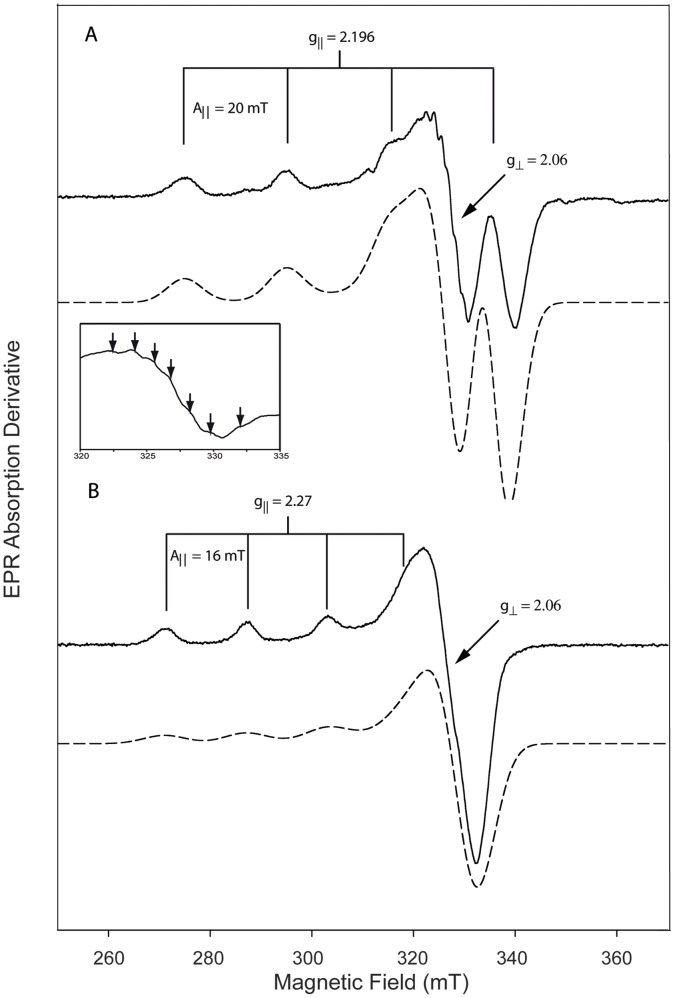
Analysis of the two major EPR signals observed during titration of MopE* with CuCl_2_. (A) The solid line shows the EPR spectrum of MopE* at 33 K with one molar equivalent of CuCl_2_ (identical to Fig. 2B, lane ii). The EPR parameters (g_⊥_, g_||_, A_||_) were read directly from the line positions, and the inset shows the superhyperfine structure observed at 77 K with one molar equivalent of CuCl_2_. Dashed line: The spectrum was simulated with the software SimFonia using Lorenzian/Gaussian ratio of 1, and line widths 6.8 mT, 7.2 mT and 5.2 mT with g = 2.197, 2.06 and 2.04, A**_||_**_Cu_ = 20 mT (B) The solid line corresponds to the difference spectrum obtained when MopE* with one molar equivalent of CuCl_2_ (Fig. 2B, lane ii ) was subtracted from MopE* with two molar equivalents of CuCl_2_ (Fig. 2B, lane iii). The EPR parameters (g_⊥_, g_||_, A_||_) were read directly from the line positions, and the spectrum was simulated (dashed line) using Lorenzian/Gaussian ratio of 1, and line widths 7.2 mT, 7.2 mT and 8.2 mT with g = 2.27, 2.06 and 2.06, A_||Cu_ = 16 mT.

The EPR spectrum recorded after addition of one molar equivalent of Cu(II) to MopE* is characteristic of a non-blue type 2 Cu (II) coordination environment with a single set of Cu(II) hyperfine lines with well resolved nuclear spin I = 3/2 splitting (Fig. 2B line ii and Fig. 4A), and since we could not observe a Cu(II) EPR signal in our control spectrum of MOPS buffer with 1 mM CuCl_2_, this signal is most likely specific for Cu(II) binding to MopE*. Integration of the spectrum revealed that 0.9 molar equivalents of Cu(II) was EPR active and bound to MopE* (Fig. 2B inset). After addition of two molar equivalents of Cu(II) to MopE* a second overlapping spectrum with characteristics of a non-blue type 2 Cu (II) coordination environment (Fig. 2B line iii and Fig. 4B) is emerging, and increasing in intensity after addition of 4 molar equivalents of Cu (II) (Fig. 2B line iv). Only a slight increase in the intensity is observed at 8 molar equivalents (Fig. 2B line v) suggesting that binding of Cu(II) to MopE* is approaching near saturation after addition of between 4 and 8 equivalents of Cu(II). Integration of the latter three spectra revealed that 1.4, 2.1, and 2.2 molar of Cu(II) was bound to MopE* (Fig. 2B inset) after addition of 2, 4 and 8 equivalents of Cu(II), respectively, and are probably in equilibrium with EPR silent aqua copper complexes. Visible inspection of the lowest magnetic field envelopes obtained after addition of 4 and 8 molar equivalents of CuCl_2_ (Fig. 2B, line iv–v), demonstrated broad possibly adventitious binding of Cu(II) ions when compared to 2 mol equivalents of CuCl_2_. The latter signal may possibly represent a third Cu(II) species (Fig. S2), explaining why complete saturation of Cu(II) binding to MopE* is not observed after addition of between 4 and 8 molar equivalents of Cu(II). Due to the corresponding low binding affinity, this third Cu(II) binding species is most likely of no, or low, relevance to the biological function of the MopE protein.

Both of the Cu(II) signals (Fig. 4A and B) observed are of the (nearly) axial type [37], showing a major axial derivative signal to higher field at g_⊥_ and a weaker derivative signal to lower field at g_||_ with higher g value. Simulation of the spectra shows that the stronger binding Cu(II) species in Fig. 4 A has g = 2.196, 2.06 and 2.04 and copper A_||_ = 20 mT and the resolved hyperfine components along g_⊥_ suggests the presence of three or four nitrogen donors in the copper coordination. Including the values obtained from the spectrum obtained at 77 K (see inset) in the EPR envelope simulation, resulted in a fit resembling the experimental data.

The second Cu(II) species (Fig. 4B) has g = 2.27, 2.04 and 2.04 and copper A_||_ = 16 mT. The two different Cu(II) EPR signals did not show any major difference in their microwave power-saturation behaviour (1 microW to 10 milliW), neither at 33 K nor at 77 K, indicating that the two different Cu(II) were located far apart (>10 Å) from each other in space (data not shown). The present system can therefore be described as containing two independent S = ½ spin centres.

**Figure 5:**
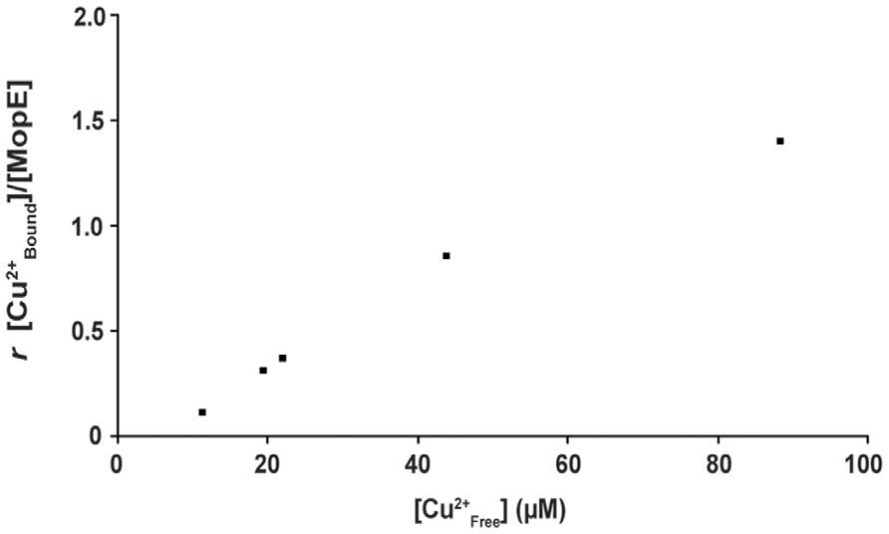
Binding of Cu^2+^ to MopE under equilibrium conditions. (A) MopE (10 µM, 500 µl) was dialysed overnight at 4°C against 100 ml of 20 mM Tris pH 7.5, 80 mM NaCl and 1 mM CaCl_2_ containing from 0 to 100 µM CuCl_2_. MopE bound Cu^2+^ was determined by ICP-MS (subtracting the Cu(II) concentrations inside and outside the dialysis cassette). The molar ratio (*r*) of bound Cu(II) to MopE* has been plotted against the concentration of CuCl_2_ in the dialysis buffer. The data were adjusted for copper bound to MopE* at no addition of CuCl_2_.

The Cu(II) binding capacity of MopE*, was also investigated under equilibrium conditions by dialysing purified protein against a Tris buffer (20 mM Tris pH 7.5, 80 mM NaCl and 1 mM CaCl_2_) with various concentrations of CuCl_2_ between 0 and 100 µM. The amount of Cu(II) bound to MopE increased when more than 10 µM CuCl_2_ was included in the dialysis buffer, and saturation was not achieved at 100 µM. The binding data were consistent with binding of Cu(II) to more than one binding site per MopE molecule, in agreement with the EPR titration data. The binding constant of the strongest Cu(II) binding was estimated to be in the micromolar range. (Figs. 5 and S3). Although saturation of Cu(II) binding to MopE was not apparent in the dialysis experiments, Scatchard plot analysis of the binding data (Fig. S3) suggests that there exist 2 Cu(II) binding sites per MopE molecule, which is consistent with the EPR titration data.

### Specific Retention of MopE* on Cu(II) Charged Columns; Amino Acid Residues at the N-terminal End of MopE* may Contribute to the Binding of Cu(II) Ions

**Figure 6:**
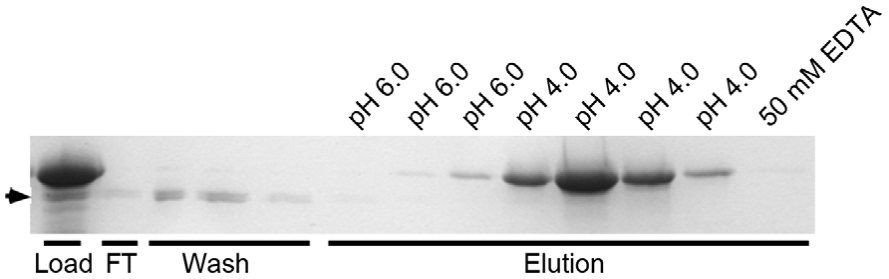
Retention of MopE* on Cu(II) charged IMAC columns. 10% SDS-PA gels containing samples from Cu(II) affinity chromatography columns; application at pH 6.8, elution performed with pH 4.0 Each eluted fraction (corresponding to one column volume = 0.5 ml) was analyzed The lanes show the load to the column (Load), the flow-through (FT), the pH 6.8 washes (Wash), the pH 6.0 elutions (pH 6.0), the pH 4.0 elutions (pH 4.0), and finally the EDTA-stripping of the column (50 mM EDTA). Application and washing was performed using 20 mM sodium phosphate buffer pH 6.8, containing 0.5 M NaCl. Elution was performed using 20 mM sodium phosphate buffer containing 0.5 M NaCl and 1 mM CaCl_2_, pH 6.0 and 4.0, respectively. The protein band indicated with an arrow represents MopE*^−24^ (see Fig. 1).

Metal binding studies were conducted using immobilized metal affinity chromatography (IMAC). MopE* could bind to a column charged with Cu(II) ions (Fig. 6), which is consistent with both the EPR and equilibrium dialysis data. MopE*did not bind to IMAC columns charged with either Fe(II), Fe(III), Ni(II), Zn(II), or Co(II), suggesting that the binding is specific for Cu(II). The bound protein could be eluted using either pH 4.0 or 100 mM imidazole (Fig. 6), respectively). Interestingly, a MopE* degradation product (indicated with an arrow in Fig. 6) lacking the first 24 N-terminal amino acid residues was unable to bind to the Cu(II) charged column, which may suggest that residues within this region are involved in coordinating the Cu(II) ions.

## Discussion

In this communication, we demonstrate that the *M. capsulatus* secreted protein, MopE*, binds Cu(I) ions in a high affinity site and (at least) two Cu(II) ions bind in weak(er) affinity sites.

The *mope* gene is expressed under limited copper conditions [19], and MopE* is secreted into the growth medium as the 336 C-terminal amino acids domain of MopE^C^ (see Fig. 1 for the structural organization of MopE). After prolonged storage in buffer solution, 24 amino acids were eventually lost from the N-terminus of MopE* protein giving MopE*^−24^ (Figs. 1 and 6), and similarly, the 46 N-terminal amino acids were invisible in the electron density maps, suggesting that the N-terminal region is cleaved off during the crystallization process, giving MopE*^−46^ [20] (Fig. 1). This is important to keep in mind when comparing the data presented in the present work to those found with the crystallized MopE* protein. During handling of MopE* in solution, the presence of CaCl_2_ stabilized the protein sufficiently for the time needed to perform the experiments presented in this communication.

A competition experiment using Bathocuproine, indicated that the copper ion identified in the crystal structure of MopE* was strongly bound to the protein (*K*_d_<10^−20^M) [20]. In the present study, EPR and X-ray absorption spectroscopy (XAS) were performed in order to obtain information about the oxidation state of the bound copper [37], [38], [39]. Taken together, the EPR (Fig. 2) and the XANES results (Fig. 3) verified that the copper bound in a high affinity mode is Cu(I) (Fig. S1), both when the protein is in a soluble and full-length form (MopE*) as well as after crystallization (MopE*^−46^) (see Fig. 1).

In addition to the strong Cu(I) binding site, the EPR analyses after addition of CuCl_2_ indicated that MopE* has two additional Cu(II) binding sites (Fig. 2B and 4A–B). The X-band EPR measurements gave information on the binding of these two additional Cu(II) ions as well as on the geometry of the binding sites.

Under the conditions used for the EPR titrations in this report it is likely that the :”free” Cu(II) ions will be in competition between binding site(s) on the MopE* (EPR active) and the sparingly soluble Cu (II) hydroxide (EPR silent), and it is thus difficult to estimate an accurate dissociation constant for Cu binding to MopE* based on the EPR data alone. However, the tabulated solubility product data for Cu(II) hydroxide is approximately 10^−20^, suggesting that the Cu(II) binding sites on MopE* must be quite strong which is consistent with the equilibrium dialysis data (discussed below). Since Cu(II) hydroxide only has limited solubility the conditions used during the incubation time may also affect the resultant spectra. However, similar Cu(II) binding characteristics are observed in the equilibrium dialysis experiments (discussed below) indicating that the EPR conditions used in this report are adequate for studying Cu(II) binding to MopE*.

The two different EPR species with ratio 1/1 per protein molecule indicated two different mononuclear Cu(II)-sites. The estimated g and A_||_ values for both Cu(II) species (Fig. 4) are typical of so called non-blue type II Cu(II) centres (also called normal Cu(II) complexes) [40], [41], [42], [43], [44], [45], [46]. Non-blue type II Cu(II) centres have in general square planar geometry with nitrogen and oxygen as coordinating ligands. This suggests that Cu(II) does not bind to the same site as Cu(I), which has a trigonal planar arrangement [20], and is also in line with the EPR analysis of the protein in crystal form (MopE*^−46^) which does not bind Cu(II). The EPR signals and absence of strong blue light absorption exclude Type I EPR active centre [37], [47]. The first binding of Cu(II) forms the species in Fig. 4A, which exhibits relative low g_||_ value and high A_||_ value for Cu(II) in proteins. Such EPR envelope (g_|| = _2.196 and A_||_ = 20 mT) is quite unusual and bears similarities with that observed in the (ethylenediamine)_2_-Cu(II) and related complexes [44], [45] while bovine serum albumin Cu(II) at pH 9.2 has even larger A_||_ value and lower g_||_ value [44]. The small distorted rhombic splitting along the g_⊥_ value from the purely axial case (g = 2.196, 2.06 and 2.06) to our measured (g = 2.196, 2.06 and 2.04) in species presented in Fig. 4A can be observed in other type II proteins, as for instance in the T2 centre of laccase [38]. The g_||_ and A_||_ parameters are in agreement with ligations to 4N, 3N1O or 2N2O [43], and the direct interaction with three or four different nitrogen nuclei is supported by the observation of a resolved superhyperfine structure in the g_⊥_ region of the EPR spectrum after addition of one equivalent of copper (Fig. 4 inset), similarly to what is found for the mono-nuclear protein Cu(II) sites in e.g. particulate methane monooxygenase [38], [41]. After addition of two equivalents of copper (Fig. 4B), the majority species g_||_ and A_||_ parameters are in agreement with ligations to 4N, 3N1O, 2N2O and 1N3O [43] and it exhibits quite normal g_||_ and A_||_ values (2.27 and 16 mT) for type II in proteins [37], [41], [48].

From our data, we cannot exclude the possibility that the protein interacts at very low affinity with Cu(II) to make a complex generating a third weak and broad EPR active Cu(II) signal, observed after addition of 4 and 8 molar equivalents of CuCl_2_ (Fig. 4C).

Binding of Cu(II) to MopE* was also investigated by equilibrium dialysis and IMAC binding. The equilibrium dialysis data were consistent with Cu(II) binding to more than one site (Fig. 5 and S3). Estimation of the apparent binding constant to the strongest site suggests a K_D_ in the micromolar range, which is consistent with the estimation from the EPR experiment (Fig. 2. insert), and is similar to the Cu affinities observed for many copper chaperones [49], [50], [51], and also that observed for methanobactin from *M. capsulatus* [9]. However, the K_D_ values reported for copper-protein binding affinities for many systems are in general very variable. The problem of scattered copper binding affinities reported for metallo-chaperones like Atox1 has recently been addressed [52]. The use of a Tris buffer system in a copper-protein study has a reducing effect on the binding affinity [53], [54], since Tris is a potential ligand for metal ions. The affinities for Cu(II) binding to MopE* estimated in this report therefore represents minimal values since the copper chelating effect of Tris should be considered when calculating a “true” K_D_ [53], [55], [56]. Overall, our data indicates that at least binding of the first higher affinity Cu(II) ion to MopE* may be of biological relevance.

It is interesting that MopE* in some respects can be compared to copper chaperones, such as CopC, which binds one Cu(II) and one Cu(I) on each side of the protein [57]. CopC is proposed to be a periplasmic Cu chaperone involved in copper detoxification [58], and it is tempting to speculate that a function of MopE is to act as a copper chaperone on the cell surface.

IMAC binding studies (Fig. 6) revealed that MopE*, but not MopE*^−24^ could bind specifically to a Cu (II) charged column, which strongly suggests that residues in the N terminal region of MopE* are involved in coordinating the two Cu(II) ions. The N terminal region of MopE* is not visible in the electron density maps of the MopE* crystals, and may explain why additional copper binding sites were not observed in the density maps when MopE* was co-crystallised with excess CuSO_4_ [20]. The truncated part of MopE* (missing in MopE*^−24^) contains multiple aspartates but no histidines: ^205^GLDTLDRDGDGSTADADCNDFAPT^228^. Carboxylate moieties are known to participate in coordination of Cu(II) ions in other proteins [59]. Coordination to cysteine is not supported by the EPR results, and the histidine(s) possibly involved, as indicated from the EPR data and from the binding of MopE* to the Cu(II)-affinity column (Fig. 6), must therefore be located elsewhere in the protein sequence. There are 7 histidine residues in MopE* (His26, His132, His164, His203, His208, His222 and His263), but analysis of the structure shows that only 4 of them His132, His203, His208 and His263 have a surface exposed side chain. His132 and His203 are involved in coordinating the Cu(I) ion and are probably not involved in Cu(II) binding. His208 and His263 are located in relative close proximity to the N-terminus in the MopE* crystal structure (Gly47) and could possible be involved in coordinating Cu(II) along with other residues in the flexible and disordered N-terminal region. His26 is located in the flexible disordered N-terminal region of MopE* and could also be involved in coordinating Cu(II). However, it is possible that the NH_2_ terminus and/or deprotonated amides [60] from the peptide backbone of MopE* are involved in coordinating Cu(II), which would also be consistent with the superhyperfine structure observed in the EPR spectrum of MopE* at 77 K with one molar equivalent of CuCl_2_ (Fig. 4A inset). The identification of the Cu(II) binding sites needs further investigations and should preferably be performed on both MopE* and MopE^c^.

The X-ray diffraction and mass spectrometry data on MopE* showed a unique kynurenine-containing copper-binding site [20]. The conversion of tryptophan to kynurenine takes place specifically in *M. capsulatus* and appears to be a prerequisite for Cu(I)-binding in wild-type MopE, and is thus related to the biological function of the protein [20]. A tryptophan residue in the vicinity of a bound copper ion appears to affect their respective redox properties [61]. Importantly, it would appear that the oxidation of tryptophan is not coupled to the binding of Cu(I) since the percentage of these Cu(I)-binding sites in MopE* actually containing Cu(I) depended largely on the copper content of the growth medium, whereas the tryptophan in question was completely oxidized in the crystal (20). Following purification, MopE* was unable to further bind copper in a strong Cu(I) binding mode, i.e. any additionally bound Cu is either not retained during purification, or it is released during dialysis against copper free buffers (this publication and ref 20). A possible explanation for this may be that the binding of Cu(I) takes place when the protein is in an partly unfolded form and/or when interacting with a partner protein. Following (re)folding of the MopE* domain, the Cu(I) binding site may be protected/shielded by the N-terminal 46 amino acids that are missing in the crystal form of the protein; this flexible and “unstructured” sequence may form a lid over the Cu(I) site barring further copper binding to this site. The loss of such a lid function would explain why the occupancy of copper increased from about 65 to near 100% when co-crystallizing (Cu-MopE*) with CuSO_4_ (20), since in the crystal, the copper-binding site is open to the solvent (20). The addition of copper to the crystallization conditions had minor effects on the binding distances (ref 20); in particular, the electron density was still well-defined around the binding histidine imidazoles (His132 and His203) and the oxidized Trp130, but less defined between the copper atom and the water molecule. This may suggest that Cu(II) could now be bound to unoccupied histidines (His132 and His203) in the crystals of MopE* (See also reference 20 for further discussion).

Whether or not MopE has a direct role in the reduction of the copper ion found in the Cu(I) binding site remains to be elucidated. It is interesting, however, to note that there is an abundance of C-type cytochromes on the surface of *M. capsulatus* [62], [63], which, with the exception of the dissimilatory metal-reducing bacteria, are not commonly observed in bacteria [64]. Several of these surface-exposed cytochromes showed a fine-tuned copper-regulated expression between 0 to 1.6 µM copper in the growth medium, i.e. in the concentration range where MopE is expressed [2], [16]. An inference is that the surface-located cytochromes act as metal-reductases, perhaps converting cupric to cuprous ions. Copper ions both in reduced and oxidized form are imperative for the function of pMMO, the key metabolic enzyme of the methanotrophs [5], and it is intriguing that MopE* is being expressed just prior to the switch from a Cu-dependent (pMMO) to a Cu-independent (sMMO) metabolism [65], [66].

It would appear reasonable, in environments where copper bioavailability is limited, that cells that are able to express a high affinity uptake system have a competitive advantage over cells that do not [1]. Methanobactin isolated from *M. capsulatus* has substantially lower affinity for copper than methanobactin isolated from *M. trichosporium* OB3B [9], having dissociation constants in the order of 10^−5^ to 10^−6^ M and about 10^−16^ M, respectively [10], [67]. Thus the affinity of Cu(II) to MopE* is of the same order as the binding to *M. capsulatus* methanobactin, whereas the very high Cu(I) affinity of MopE* is more in the order exhibited by the *M. trichosporium* OB3B methanobactin. In this context, it is important to note that the latter bacterium does not express MopE. In line with their respective apparent binding constants, it was observed that, when grown under copper limited conditions, *M. capsulatus* methanobactin was isolated from the medium without bound copper [13] whereas MopE* is isolated with bound copper [20]. Taken together, the findings give support to the hypothesis that MopE is acting as a species specific and environment depending copper uptake system, presumably utilizing both its high (Cu(I) and low Cu(II) affinity properties. In particular, MopE* may possibly act as a copper chaperone that delivers both Cu(I) and Cu(II) to other proteins under Cu limiting conditions.

## Supporting Information

Figure S1 Illustration of the binding site for reduced copper in MopE*. Cu(I) is coordinated by His132, His203, Kynurenine130 and a solvent molecule in a tetragonal arrangement. The geometry and distances (in Angstrom) is obtained from PDB entry 2VOV.
(TIF)

Figure S2 Analysis of the weak third EPR signal observed after addition of 4 and 8 molar equivalents of CuCl2 to MopE*. The solid line corresponds to the difference spectrum obtained when MopE* with 8 molar equivalents of CuCl_2_ (Fig. 2B, lane v) was substracted from MopE* with 4 molar equivalents of CuCl_2_ (Fig. 2B, lane iv). The spectrum was simulated (dashed line) using Lorenzian/Gaussian ratio of 1, and line widths 5.0 mT, 6.5 mT and 5.0 mT with g = 2.305, 2.060 and 2.064, A||Cu = 15.8 mT.
(TIF)

Figure S3 **Scatchard plot analysis of the equilibrium dialysis data.** The dissociation constants (K_D_) were determined from the reciprocal of the linear slopes (dashed lines). *r* is to the molar ratio of bound Cu(II) to MopE*. The analyses indicate two distinct affinities for Cu(II).
(TIF)
