# Supplementary figures and images for: The Methylococcus capsulatus (Bath) Secreted Protein, MopE*, Binds Both Reduced and Oxidized Copper
Source: PLoS One. 2012 Aug 20;7(8):e43146. doi: 10.1371/journal.pone.0043146 (PMC3423442; doi:10.1371/journal.pone.0043146)

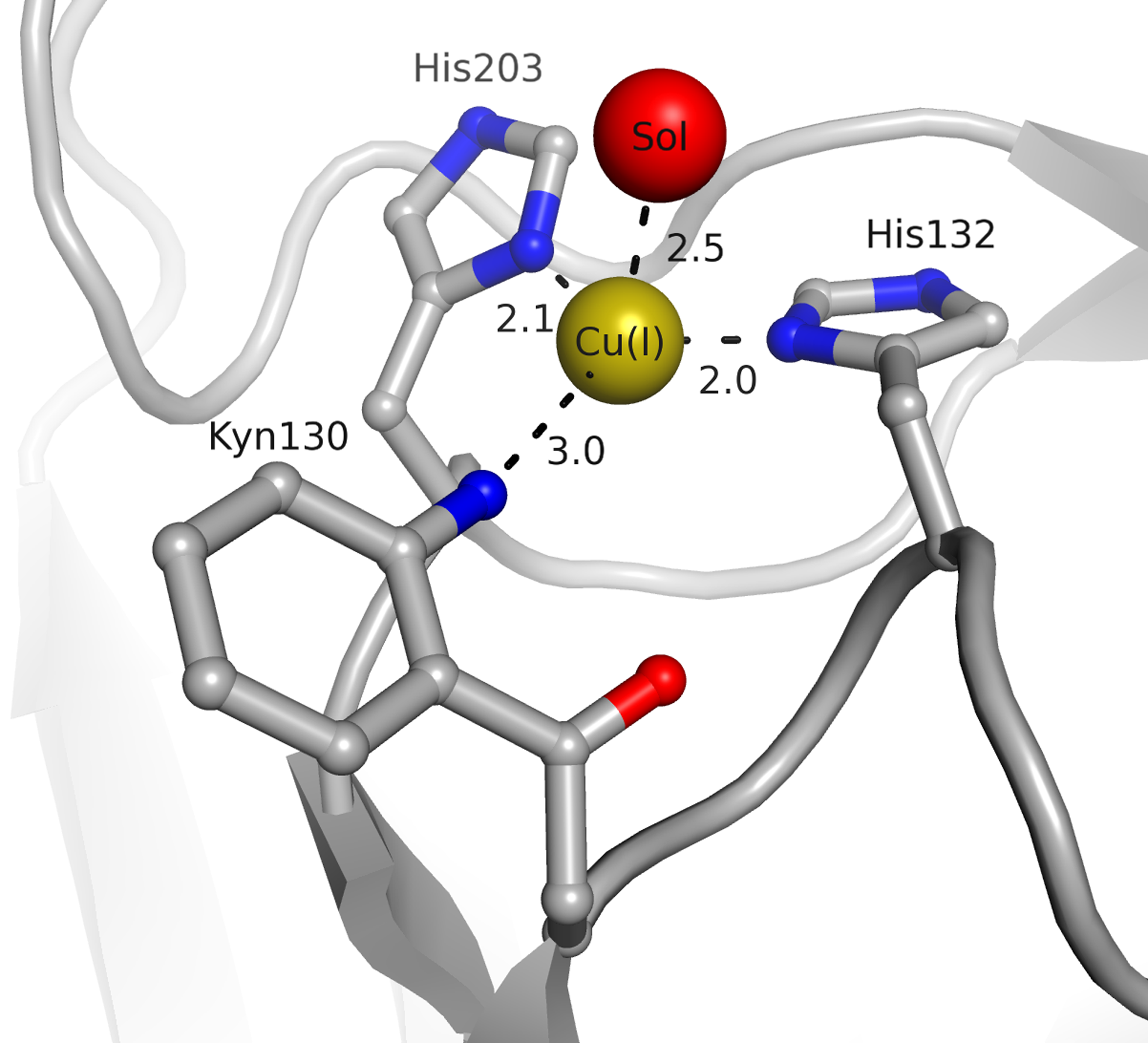

Supplement: Figure S1 — Illustration of the binding site for reduced copper in MopE*. Cu(I) is coordinated by His132, His203, Kynurenine130 and a solvent molecule in a tetragonal arrangement. The geometry and distances (in Angstrom) is obtained from PDB entry 2VOV. (TIF) [file pone.0043146.s001.tif]

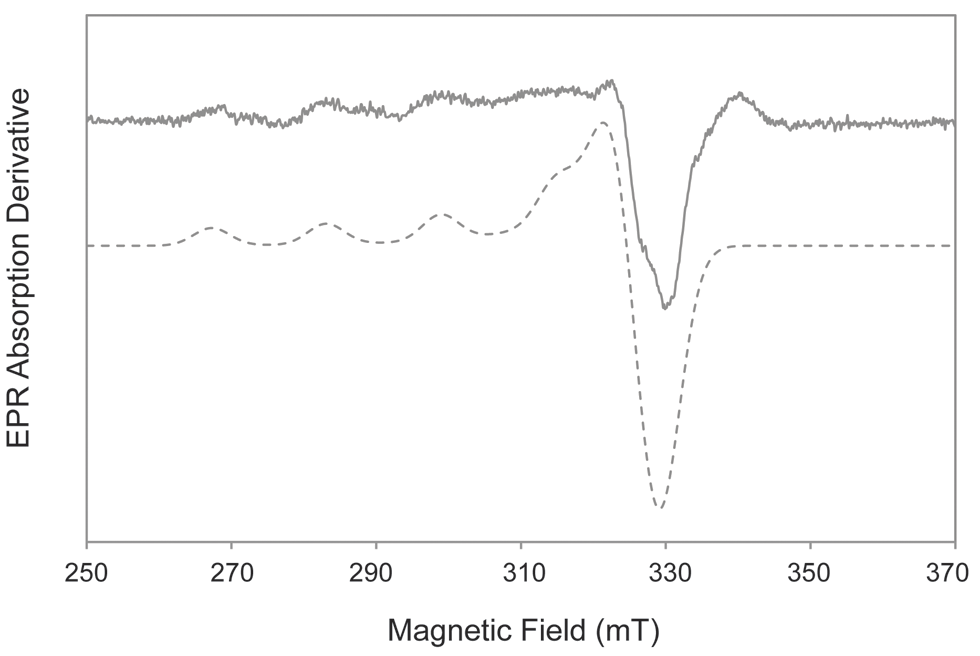

Supplement: Figure S2 — Analysis of the weak third EPR signal observed after addition of 4 and 8 molar equivalents of CuCl2 to MopE*. The solid line corresponds to the difference spectrum obtained when MopE* with 8 molar equivalents of CuCl2 (Fig. 2B, lane v) was substracted from MopE* with 4 molar equivalents of CuCl2 (Fig. 2B, lane iv). The spectrum was simulated (dashed line) using Lorenzian/Gaussian ratio of 1, and line widths 5.0 mT, 6.5 mT and 5.0 mT with g = 2.305, 2.060 and 2.064, A||Cu = 15.8 mT. (TIF) [file pone.0043146.s002.tif]

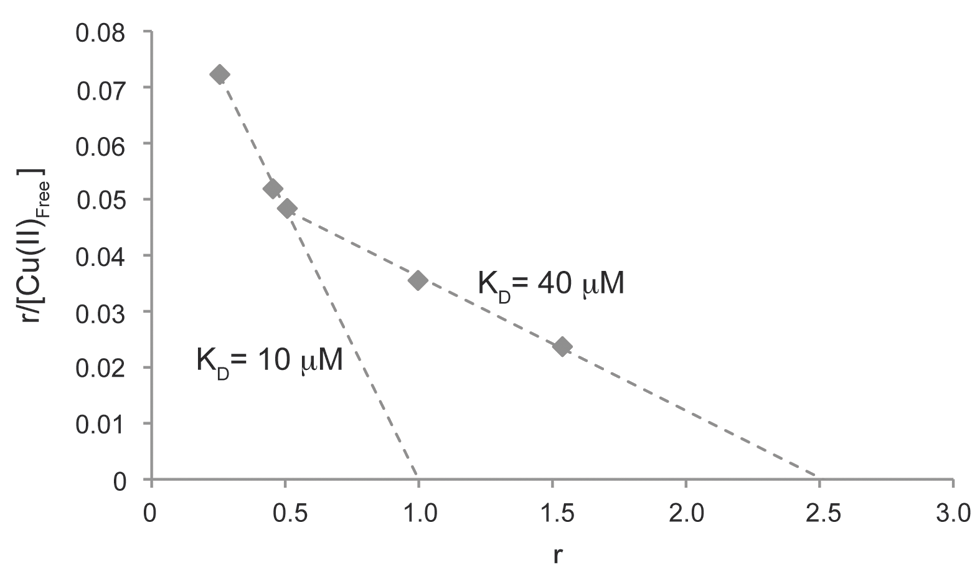

Supplement: Figure S3 — Scatchard plot analysis of the equilibrium dialysis data. The dissociation constants (KD) were determined from the reciprocal of the linear slopes (dashed lines). r is to the molar ratio of bound Cu(II) to MopE*. The analyses indicate two distinct affinities for Cu(II). (TIF) [file pone.0043146.s003.tif]
